# Aggregate Sampling to Detect Pathogens and Antimicrobial Resistance Genes Associated with Bovine Respiratory Disease in US Feedlots: A Pilot Study

**DOI:** 10.3390/vetsci12030244

**Published:** 2025-03-04

**Authors:** Erin Jobman, Brian Vander Ley, John Dustin Loy, Duan Sriyotee Loy, Nathan Meyer, Dan Thomson, James Lowe, Shane Terrell

**Affiliations:** 1 Production Animal Consultation, 307 S. Main, Scott City, KS 67871, USA; ejobman2@illinois.edu (E.J.); dan.thomson@pacdvms.com (D.T.); jlowe@illinois.edu (J.L.); 2 Department of Veterinary Clinical Medicine, University of Illinois at Urbana-Champaign, 2001 S. Lincoln Ave., Urbana, IL 61802, USA; 3 School of Veterinary Medicine and Biomedical Sciences, University of Nebraska-Lincoln, 1880 N 42 St., Lincoln, NE 68583, USA; bvanderley2@unl.edu (B.V.L.); jdloy@unl.edu (J.D.L.); dloy2@unl.edu (D.S.L.); 4 Boehringer Ingelheim Animal Health USA, 3239 Satellite Blvd NW, Duluth, GA 30096, USA; nathan.meyer@boehringer-ingelheim.com

**Keywords:** bovine respiratory disease, aggregate diagnostics, epidemiology

## Abstract

Bovine respiratory disease (BRD) is the leading cause of feedlot morbidity and mortality. Field diagnosis is often limited to visual examination as available diagnostics reflect individual animals only and require labor, animal restraint, and time. Aggregate sampling techniques are valuable tools in other species but are lacking in the beef industry. This pilot study investigates the plausibility of using the water trough as an aggregate sample substrate in pens of confined cattle. Water and swab substrates from ten water tanks were collected at ten sampling events. Samples were subjected to a multiplex PCR to detect viruses, bacteria, and antimicrobial resistance (AMR) genes associated with BRD. Viral and bacterial PCR detections differed significantly among morbidity classes (Fisher’s exact *p* = 0.0139 water; *p* = 0.0222 swab). The overall kappa and Percent Positive Agreement were 0.72 and 84.01% among sample substrates. Bayesian latent class analysis was used to estimate the probability of detection. Viral and bacterial organisms reached peak sensitivity (21–79%) on days 4–21 and peak specificity (44–79%) on days 42–56. All AMR genes’ sensitivity and specificity remained relatively constant throughout the sampling period.

## Simple Summary

This pilot study investigated a population-based sampling technique to detect pathogens associated with bovine respiratory disease. The US feedlot industry typically manages cattle in groups, at the lot, or pen level; however, current diagnostics reflect individual animals only. Population-based sampling could be advantageous to the cattle industry because it is non-invasive, reduces handling stress, and improves safety for the animals and personnel involved. The primary objective was to investigate the presence of pathogens in water troughs from pens of confined cattle. Viruses, bacteria, and antimicrobial resistance genes associated with bovine respiratory disease were successfully detected in the water tanks during the same period of conventional visual disease detection. These findings provide a foundation for population-based testing to better manage current bovine disease challenges and could be pivotal in the detection of emerging diseases.

## 1. Introduction

Bovine respiratory disease (BRD) is the leading cause of morbidity and mortality in US feedlots, developing from intricate interactions between pathogens, stress, and host factors [1,2,3]. This complex syndrome contributes approximately 70–80% of total morbidity and 10–50% mortality in US feedlots [2]. Cattle are exposed to varying degrees of stress surrounding feedlot entry including weaning, comingling, dietary transitions, and transportation. Viruses commonly isolated from affected animals include bovine viral diarrhea virus (BVDV), bovine respiratory syncytial virus (BRSV), and bovine herpes virus 1 (BHV-1). Bovine coronavirus is known to cause calfhood diarrhea; while its role in BRD is not fully understood, research supports a trophism for respiratory tract epithelium in cattle [4,5]. Four bacteria typically isolated from morbid animals and respiratory tissues of deceased animals include *Mannheimia haemolytica*, *Pasteurella multocida*, *Histophilus somni*, and *Mycoplasma bovis* [2].

At this time, BRD diagnosis is primarily focused on a visual exam identifying signs of depression, inappetence, and abnormal respiratory rates; however, cattle are prey species and will attempt to mask illness from predators [1,2,3]. Antemortem diagnostic methods, such as nasal swabs, bronchoalveolar lavage, and transtracheal washes, reflect individual animals and require labor, time, and animal restraint.

Aggregate sampling allows for a concurrent collection of multiple individuals, providing a broad overview of health trends. In veterinary medicine, this approach minimizes the need for invasive procedures on individual animals, thus reducing stress, ensuring safety, and improving animal welfare. Swine veterinarians frequently use aggregate samples, such as oral fluids, as an effective diagnostic tool in the detection and surveillance of respiratory viruses [6,7,8]. Similarly, viruses and bacteria can be detected by sampling bulk milk tanks in dairies [9,10]. In human medicine, wastewater is a valuable surveillance substrate to regionally detect illicit drug use and infectious diseases, such as COVID-19 [11,12]. Additionally, environmental samples, such as bioaerosols, have been utilized for respiratory virus detection in swine and poultry farms, and even military barracks [13,14,15]. Pen-level sampling in confined cattle was previously explored using ropes and fecal samples to detect *Escherichia coli* and *Salmonella spp*. [16,17]. However, there are currently no aggregate sampling techniques to detect bovine respiratory disease [8,18].

In the US feedlot industry, management decisions are often made at the lot or pen level. The ability to detect pathogens at the pen level could lead to improved accuracy in BRD case definition, enhanced precision of antimicrobial therapies, and superior treatment outcomes. Additionally, the success of an aggregate diagnostic tool could be critical in the detection of emerging viruses, foreign animal disease, and zoonotic pathogens.

The objectives of this pilot study are to determine if the water trough can serve as an aggregate sample substrate to detect pathogens associated with bovine respiratory disease and to describe the viral, bacterial, and antimicrobial resistance profiles over time.

## 2. Materials and Methods

### 2.1. Experimental Design

Ten pens from two US commercial feedlots were enrolled in an observational pilot study. Five pens were sampled at each site; the same pens were sampled 10 times. All pens were of similar size and design; average pen dimension was approximately 35,000 ft^2^ (3252 m^2^). All pens utilized identical concrete water troughs, hereafter referred to as tanks. Commercial feedlots typically utilize automatic water tanks with an adjustable float valve system to ensure an adequate water supply is available at all times. Each pen had its own tank and did not share water sources with adjacent pens. All water tanks had a 70-gallon capacity (265 L). Eligible pens were selected with the assistance of feedlot managers to represent diversity in respiratory disease risk assessment (low to high) at procurement. Risk assessment was based on the animals’ history prior to feedlot placement including source, distance traveled, and management records [1,3]. Cattle husbandry practices were equivalent for all pens enrolled. Daily visual assessment of health was completed by pen riders, typical for the US feedlot industry. Health records from the first 100 days on feed (DOF) were obtained from each pen. All pens received identical vaccines at processing, including a modified-live, parenteral respiratory vaccine and a killed clostridial vaccine. Pens were filled between 8 November 2023 and 29 November 2023.

### 2.2. Sample Collection and Processing

Water tank samples were collected at 10 sampling points through the first 60 days on feed. Two samples were collected at each sampling event including 50 mL of water and a 2 × 2″ absorbent, cotton swab of the tank’s water–air interface. The water sample was collected by skimming the surface of the water tank. This technique is derived from preliminary data [19] and commonly includes organic, particulate matter such as feed particles and nasal secretions. These samples were immediately stored on ice and frozen to −20° C within 2 h of collection. The swab sample was collected by dragging the swab in a serpentine pattern across the water–air interface of the tank. These samples were immediately submerged in PrimeStore MTM, a preservation media to stabilize RNA, DNA, and mRNA [20], and stored at room temperature.

Samples were collected on the following days: 0, 4, 7, 14, 21, 28, 35, 42, 49, and 56. This sampling window was selected to capture stressful dynamics surrounding placement into the feedlot, processing events, and initial treatments for BRD [2,3]. Further, the study period represents opportunities for clinical interventions to potentially change the outcome of the pen.

On day zero, samples were collected while the pen was empty. Tanks are normally cleaned between groups of cattle by emptying the tank, scrubbing with a brush ± chlorine-based disinfecting agent. To completely disinfect a concrete water tank, implementing dry time behind a disinfecting agent would be required [21]; however, this is not typical nor practical in US feedlots. For observational study purposes, tanks were cleaned per feedlot standard operating procedures and allowed to refill with water prior to day 0 sample collection. Throughout the feeding period, standard practice for both participating feedlots included weekly cleaning of the water tanks. Sample collection occurred 3–4 days following the weekly tank cleaning.

Both samples were subjected to total nucleic acid extraction followed by multiplexed real time quantification polymerase chain reaction (RT-qPCR) assays at the Nebraska Veterinary Diagnostic Center. The assays are designed to detect the following viruses, bacteria, and antimicrobial resistance (AMR) genes associated with BRD: bovine viral diarrhea virus (BVDV), bovine coronavirus (BCV), bovine respiratory syncytial virus (BRSV), and bovine herpes virus type 1 (BHV-1), *Mannheimia haemolytica*, *Pasteurella multocida*, *Histophilus somni*, *Mycoplasma bovis*, *mphE*, *erm42*, and *msrE* (macrolide resistance gene targets), and *tetR* (tetracycline resistance gene target) [22,23]. The AMR gene targets were selected for spatial and temporal consistency in BRD pathogen strain genomes and confer resistance to clinically relevant drug classes as previously described [23]. Cycle threshold (Ct) values < 40 are reported as detected for this assay.

### 2.3. Statistical Analysis

Python (version 3.9.13) was utilized for data analysis using Jupyter Notebook (version 6.4.12) as the interactive computational environment for code development and execution. Multiple data visualization and descriptive techniques were utilized to describe the results using Python’s Seaborn, Matplotlib, and SciKitLearn libraries. The kappa statistic was calculated to estimate the level of agreement between water and swab detections beyond random chance. Cohen’s suggested kappa interpretation includes values ≤ 0 as indicating no agreement, 0.01–0.20 as none to slight, 0.21–0.40 as fair, 0.41– 0.60 as moderate, 0.61–0.80 as substantial, and 0.81–1.00 as almost perfect agreement [24]. The Percent Positive Agreement (PPA) was calculated by considering the number of tanks positive by both methods, divided by the average number of tanks positive by either method * 100.

Without a gold standard reference test, sensitivity and specificity were estimated for each sample technique using a Bayesian latent class analysis. In the context of this study, sensitivity and specificity reflect the probability of detection. Bayesian modeling was conducted using Stan, a probabilistic programming language in RStudio (RStudio version 4.2.1). A non-informative prior distribution was applied for sensitivity and specificity, initialized with beta (1,1) due to the nature of the pilot study. The model used 20,000 iterations in 4 chains, thinned by 5, with a warmup initiation of 5000 runs. Given the categorical nature of PCR results (detected/not detected), log probability mass functions following Bernoulli distributions were used. Posterior estimate distributions were visually assessed with area plots via Markov Chain Monte Carlo methods [25]. The model was deployed for each PCR organism detected and by each sampling day.

## 3. Results

Ten pens were enrolled and sampled in this study. Overall, the average calf weight at placement was 675.0 lbs. ± 128.1 (306.7 kgs ± 58.2). In the first 100 days on feed, the respiratory morbidity per pen ranged from 0.7–50.8% of animals affected. Total mortality ranged from 0.6–9.7%. The average number of animals per pen was 157 ± 22. The observed respiratory morbidity was further divided post hoc into three categories: low, moderate, and high for morbidity < 15%, 16 to 30%, and >31%, respectively. This division is based on the average respiratory morbidity reported in US feedlots [26]. Placement weight, respiratory morbidity, and total mortality are presented in Table 1.

**Figure 1:**
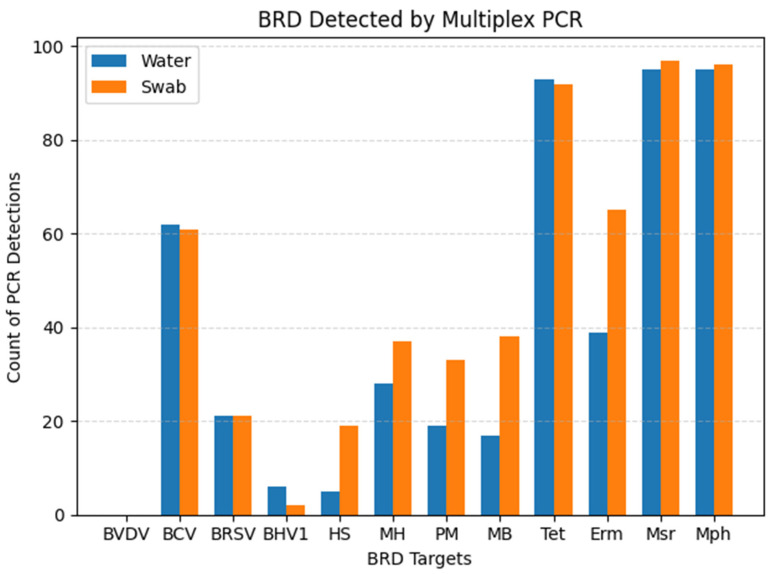
Total PCR detections per sample substrate. BVDV: bovine viral diarrhea virus; BCV: bovine coronavirus; BRSV: bovine respiratory syncytial virus; BHV1: bovine herpes virus 1; HS: *Histophilus somni*; MH: *Mannheimia haemolytica*; PM: *Pasteurella multocida*; MB: *Mycoplasma bovis*; Tet: tetracycline resistance gene; Erm: macrolide resistance gene; Msr: macrolide resistance gene; Mph: macrolide resistance gene.

All ten pens received tylosin in the feed for reduction of liver abscesses. Nine out of ten pens received chlortetracycline in the feed for the treatment of respiratory disease. Six out of ten pens received an injectable antibiotic at the time of initial processing, known as metaphylactic control of respiratory disease. Six pens were heifers only, three pens were steers only, and one pen fed a mixed-sex group. The cumulative distribution of detected viral, bacterial, and AMR genes from each sample substrate can be visualized in Figure 1.

Agreement between the sample substrates is depicted in Table 2. The prevalence of each multiplex component varies, and thus wide ranges of PPA and kappa are observed. The overall PPA and kappa are 84.01% and 0.72, respectively. Bayesian latent class analysis generated wide ranges of sensitivity and specificity (Table 3). The highest mean sensitivity was observed for BCV, BRSV, and all four AMR genes. Viral and bacterial organisms reached peak sensitivity values on days 4–21 and peak specificity values on days 35–56 (Supplementary Tables S1 and S2). For example, BCV reached 79% sensitivity on day 7 and a specificity of 55% on day 42. *Mannheimia* reached a peak sensitivity of 67% on day 4 and a peak specificity of 79% on day 56. All four AMR genes’ sensitivity and specificity remained relatively constant throughout the sampling period. BVDV was not detected in this dataset and was excluded from further Bayesian analyses.

**Figure 2:**
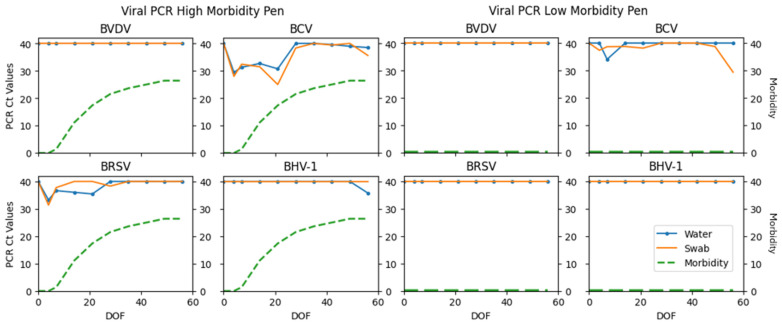
Viral PCR panel results detected from one pen with high morbidity and one pen with low morbidity. Cycle threshold (Ct) values are plotted on *y*-axis; values < 40 are reported as detected. The twin *y*-axis represents percent cumulative morbidity using the same scale. Days on feed (DOF) is plotted along the *x*-axis. BVDV: bovine viral diarrhea virus; BCV: bovine coronavirus; BRSV: bovine respiratory syncytial virus; BHV-1: bovine herpes virus 1.

**Figure 3:**
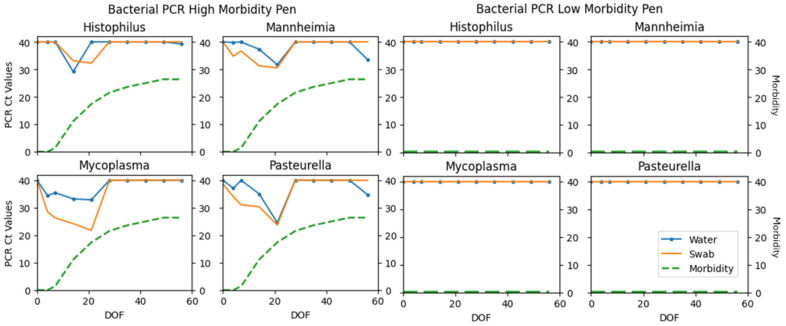
Bacterial PCR panel results detected from one pen with high morbidity and one pen with low morbidity. Cycle threshold (Ct) values are plotted on *y*-axis; values < 40 are reported as detected. The twin *y*-axis represents percent cumulative morbidity using the same scale. Days on feed (DOF) is plotted along the *x*-axis.

**Figure 4:**
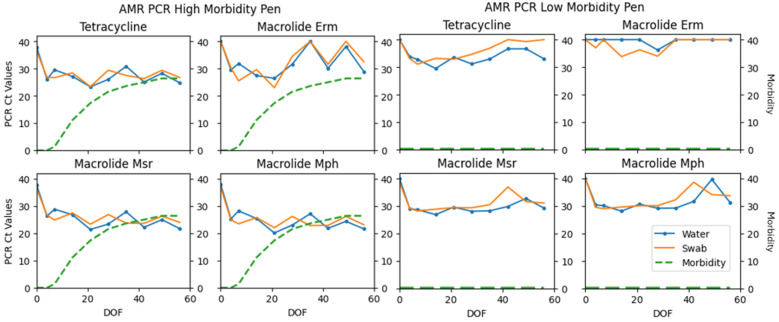
Antimicrobial resistance (AMR) gene target PCR panel results detected from one pen with high morbidity and one pen with low morbidity. Cycle threshold (Ct) values are plotted on *y*-axis; values < 40 are reported as detected. The twin *y*-axis represents percent cumulative morbidity using the same scale. Days on feed (DOF) is plotted along the *x*-axis.

Fisher’s exact test was used to determine if there was a significant association with total PCR viral and bacterial detections and observed morbidity categories. Statistical significance was declared if *p*-values were ≤0.05. A significant association was detected for sample specimens (*p* = 0.0139 water; *p* = 0.0222 swab). Differences in PCR detections between low-morbidity pens compared to a higher-morbidity pen can be visualized in Figure 2, Figure 3 and Figure 4. The same pens are represented in each figure. These figures illustrate the fluctuation in PCR cycle threshold (Ct) values across time per viral, bacterial, and AMR multiplex PCR panel. The respective cumulative pen morbidity percent is overlayed on each subplot, using the same *y*-axis scale. Minimal viral and bacteria are detected from the low-morbidity pen compared to the high-morbidity pen; however, AMR genes are detected in both pens. Overall, the moderate and high-morbidity pens displayed similar trends of pathogen prevalence and cycle threshold values.

Relationships between diagnostic results, pen demographics, and sampling day were further investigated in multiple individual analyses. To account for repeated measures, mixed-effects logistic regression was used to analyze the association between PCR detections and sampling days. Generalized linear mixed-model regression analyses were used to explore the relationship of morbidity and pen demographics. These analyses were deemed unsuitable due to small sample size, unreliable estimates of random effects, and poor model convergence.

## 4. Discussion

The results of this pilot study investigate the plausibility of using the water tank as an aggregate sample substrate in pens of confined cattle. Viral, bacterial, and AMR components of BRD were detected throughout the first 60 days on feed in varying degrees. PCR detections differed significantly among observed morbidity categories (Table 1; Fisher’s exact *p* = 0.0139 water; *p* = 0.0222 swab). Figure 2, Figure 3 and Figure 4 illustrate the comparison between a low morbidity to a high morbidity pen in relation to PCR detections from viral, bacterial, and AMR panels. In this dataset, detections of viral and bacterial pathogens occur early in the feeding period (≤21 DOF) as visually observed morbidity increases. Pathogen detections tended to plateau before decreasing to an undetectable level as the feeding period progressed (≥42 DOF) and morbidity plateaued. AMR genes tended to be detected early and remain detected throughout the sampling period. Peak sensitivity ranges (21–79%) observed early in the feeding period suggest the probability of detection is greatest from days 4–21.

The presence of viral and bacterial nucleic acids does not necessarily indicate active infections. It is possible the viral detections, apart from coronavirus, are influenced from the use of parenteral, modified live vaccines in these animals [27]. However, all pens received equivalent vaccines and the low-morbidity pens did not demonstrate similar viral detections as the high-morbidity pens. In addition, the detection of AMR genes in these samples does not necessarily indicate that they originated from BRD pathogens as the genes are potentially mobile and can be found in other organisms [23]. However, these genes are consistently found in BRD pathogens and have high predictive values in bovine lungs for the isolation of phenotypically resistant *Mannheimia* [23]. Specimens included in the multiplex PCR may or may not be representative of the true microbial dynamics in the water tanks. Water tanks have been examined in another study, which demonstrated profound diversity in bacteria and AMR profiles using whole genome sequencing [28]. The water tanks represent a focal area of the pen that each animal visits and contributes saliva and nasal secretions; however, the tank can also capture environmental dust, manure, feed particles, etc., from which the AMR genes could originate, as these genes are associated with BRD pathogens, but are not necessarily specific to them. For this reason, it is possible that wildlife, such as birds, could contribute to AMR gene detections. These factors potentially explain the AMR prevalence from the low-morbidity pens in Figure 4. Nonetheless, this study illustrates a pattern of detection, or perhaps a manifestation of stress, while clinical observations of morbidity accumulate. Further research is needed to understand the magnitude of these trends.

The agreement between the water sample and tank swab was explored with Percent Positive Agreement, kappa, and Bayesian latent class estimates of sensitivity and specificity. The overall agreement between the water and swab samples varied depending on what was detected (viral, bacterial, or AMR genes) and when. Percent Positive Agreement ranged from 17.39% to 99.48%. Kappa results ranged from 0.104 to 0.884. The overall PPA and kappa are 84.01% and 0.719, respectively. A more uniform cattle population may improve precision in these estimates.

There is no gold standard in evaluating aggregate diagnostic samples in bovine medicine [18,29]. There is also no gold standard for the clinical diagnosis of BRD [30,31,32,33]. Therefore, Bayesian latent class analysis was applied to estimate sensitivity and specificity of each substrate’s ability to detect pathogens. This methodology provides a rigorous approach to assessing diagnostic test accuracy in the absence of a gold standard [34,35], offering insights into the utility of water samples for detecting pathogens and estimating pathogen shedding levels in the field. The Bayesian latent class analysis framework allows for the estimation of test accuracy without relying on strong prior assumptions, thereby accommodating the exploratory nature of the pilot study. Sensitivity and specificity reflect prevalence, which can be dynamic over time [24]. Thus, sensitivity and specificity estimates vary across sampling days and from organism to organism; nonetheless, overlapping confidence intervals suggest comparable performance between water and swab samples.

Most published Bayesian analyses use informative, prior beta distributions based on expert panel opinions and previous studies. A previous study estimated the sensitivity of visually observed clinical illness in beef cattle to be 57.5–62.2% and specificity to be 62.7–62.9% [30]. This study also estimated the sensitivity of lung lesions at harvest to be 77.4–84.7%. These results suggest 38% of truly diseased animals are missed, and approximately 37% of calves without disease are treated as such [30]. Another study found similar results in dairy calves using thoracic ultrasound (sensitivity 79.4%; specificity 93.9%) and clinical respiratory scores (sensitivity 62.4%; specificity 74.1%) [32]. Other studies have estimated the sensitivity and specificity of visually observed clinical illness to be 27% and 92%, respectively [33].

These studies reflect the complexity of identifying BRD in the field without the aid of diagnostics. Clinical signs typically used to identify morbid animals are subjective and not pathognomonic for respiratory disease. Without pragmatic, objective criteria, such as diagnostics, accuracy in BRD case definitions and the assessment of causal agents remain challenging.

### Limitations

Given the nature of a longitudinal pilot study, many limitations exist. A robust comparison to conventional individual animal sampling, such as nasal swabs, would aid in the analysis and interpretation of these results. In addition, the sample size is underpowered to draw conclusions of the effect of viral and bacterial prevalence. Mixed models yielded unstable estimates or failed to converge, creating unreliable results. Conditional dependence may also exist that was not accounted for. Overall, sensitivity reflects the probability of detection. Further, enrolled pens were not randomly selected to intentionally include low and high-risk pens. This created variation in prevalence, which is reflected in the results. Sensitivity, specificity, and confidence intervals would likely improve with a more uniform cattle population. Additionally, any dilution effect within the tanks is largely unknown and not considered in this study. Seasonal effects and pathogen longevity are also unknown with this technique and require additional research.

## 5. Conclusions

To respond quickly to rapidly evolving morbidity scenarios and/or emergent diseases, cattle veterinarians need population-based sampling methods to provide a pragmatic, continual assessment of pens. Aggregate samples in other species are economical, convenient, and timely to detect pathogens and estimate herd prevalence. The dynamic, polymicrobial nature of BRD challenges practicality in choosing effective diagnostics. Nonetheless, we believe this methodology has significant potential to better our understanding of BRD. In addition, we believe this methodology could serve as the initial diagnostic analysis for emergent viruses and disease surveillance. Multiple follow-up studies are warranted to assess the utility of this methodology with conventional methods of disease detection. This technique requires additional research and continued development for adaptation into industry utilization.

## Figures and Tables

**Table 1:** Average and standard deviations for each category of respiratory morbidity observed.

Category	Placement Weight (lbs.)	Morbidity (%)	Mortality (%)
Low (*n* = 2)	849.5 (227.0)	2.2 (2.1)	1.1 (0.5)
Moderate (*n* = 5)	621.7 (24.4)	24.4 (5.5)	3.6 (3.9)
High (*n* = 3)	647.5 (90.5)	40.5 (9.8)	4.7 (3.9)

**Table 2:** Test agreement of PCR results for sample substrates across all time points. BRD: bovine respiratory disease; Detected (+), Not Detected (−); Percent Positive Agreement (PPA); BVDV: bovine viral diarrhea virus; BCV: bovine coronavirus; BRSV: bovine respiratory syncytial virus; BHV-1: bovine herpes virus 1; *H. somni*: *Histophilus somni*; *M. haem*: *Mannheimia haemolytica*; *P. mult*: *Pasteurella multocida*; *M bovis*: *Mycoplasma bovis*; Tet: tetracycline resistance gene; Erm: macrolide resistance gene; Msr: macrolide resistance gene; Mph: macrolide resistance gene.

BRD	Swab +/ Water +	Swab −/ Water −	Swab +/ Water −	Swab −/ Water +	PPA ^1^ (%)	Kappa ^2^ (95% CI)
BVDV	0	100	0	0	0	−1
BCV	45	22	16	17	73.17	0.30 (0.11–0.49)
BRSV	15	73	6	6	71.43	0.64 (0.45–0.83)
BHV-1	2	94	0	4	50.00	0.49 (0.06–0.91)
*H. somni*	2	79	16	3	17.39	0.10 (0.02–0.31)
*M. haem.*	24	59	13	4	73.85	0.62 (0.46–0.78)
*P. mult.*	16	66	15	3	64.00	0.53 (0.35–0.71)
*M. bovis*	16	61	22	1	58.18	0.45 (0.29–0.62)
Tet	91	6	1	2	98.38	0.78 (0.55–0.99)
Erm	36	32	29	3	69.23	0.40 (0.25–0.55)
Msr	34	2	3	1	97.92	0.48 (0.05–0.91)
Mph	95	4	1	0	99.48	0.88 (0.66–0.99)

^1^ PPA overall 84.01%. ^2^ Kappa overall 0.72 (0.68–0.76).

**Table 3:** Estimated mean sensitivity and specificity with 95% confidence intervals of each sample substrate utilizing a Bayesian latent class analysis. BRD: bovine respiratory disease; BVDV: bovine viral diarrhea virus; BCV: bovine coronavirus; BRSV: bovine respiratory syncytial virus; BHV-1: bovine herpes virus 1; *H. somni: Histophilus somni*; *M. haem: Mannheimia haemolytica*; *P. mult: Pasteurella multocida*; *M bovis: Mycoplasma bovis*; Tet: tetracycline resistance gene; Erm: macrolide resistance gene; Msr: macrolide resistance gene; Mph: macrolide resistance gene.

BRD	Sensitivity (%)		Specificity (%)	
	Water	Swab	Water	Swab
BVDV	-	-	-	-
BCV	57 (4–98)	56 (4–97)	45 (3–96)	46 (3–96)
BRSV	61 (1–99)	62 (1–99)	76 (4–100)	76 (4–100)
BHV-1	38 (1–98)	27 (1–93)	64 (2–97)	75 (7–99)
*H. somni*	18 (1–86)	31 (3–92)	82 (15–97)	69 (7–98)
*M. haem.*	23 (1–95)	31 (1–98)	40 (2–99)	30 (1–97)
*P. mult.*	53 (1–97)	68 (3–99)	80 (8–98)	69 (3–99)
*M. bovis*	48 (1–97)	73 (4–96)	82 (10–99)	64 (1–98)
Tet	98 (95–99)	98 (97–99)	77 (41–90)	81 (47–92)
Erm	86 (18–96)	87 (21–97)	65 (3–98)	63 (3–97)
Msr	94 (92–97)	96 (94–98)	74 (40–91)	78 (39–92)
Mph	97 (95–99)	98 (96–99)	76 (49–92)	80 (50–93)

## Data Availability

Data available upon request to the corresponding author.

## Supplementary Materials

The following supporting information can be downloaded at https://www.mdpi.com/article/10.3390/vetsci12030244/s1. Table S1: Bayesian latent class analysis sensitivity estimates; Table S2: Bayesian latent class analysis specificity estimates.
