# Supplementary material for: Aggregate Sampling to Detect Pathogens and Antimicrobial Resistance Genes Associated with Bovine Respiratory Disease in US Feedlots: A Pilot Study
Source: Vet Sci. 2025 Mar 4;12(3):244. doi: 10.3390/vetsci12030244 (PMC11946104; doi:10.3390/vetsci12030244)
Supplement: Supplementary file 1 [file vetsci-12-00244-s001.zip › vetsci-3467291-supplementary.pdf]

Supplementary Table S1. Bayesian latent class analysis sensitivity estimates.

		Sampling Day									
PCR	Sample	0	4	7	14	21	28	35	42	49	56
BVDV	Water	-	-	-	-	-	-	-	-	-	-
	Swab	-	-	-	-	-	-	-	-	-	-
BCV	Water	27	70	79	73	62	50	51	45	56	56
	Swab	38	78	60	67	68	50	45	45	50	62
BRSV	Water	24	47	40	51	61	21	29	24	28	25
	Swab	24	48	46	45	48	40	22	24	28	25
BHV1	Water	24	24	30	38	25	35	25	25	25	30
	Swab	25	24	23	38	25	21	25	25	25	23
HS	Water	25	28	22	29	21	21	22	22	30	33
	Swab	24	28	35	36	45	35	35	35	36	27
MH	Water	25	60	43	44	53	28	23	30	32	40
	Swab	25	60	67	62	60	41	30	36	32	21
PM	Water	24	48	32	44	43	30	23	22	32	35
	Swab	25	61	62	56	56	37	29	30	32	21
MB	Water	25	24	43	38	48	35	21	21	21	30
	Swab	24	24	56	56	61	47	40	34	40	37
Tet	Water	47	47	76	76	76	75	76	72	75	30
	Swab	47	47	76	76	76	75	76	72	75	37
Erm	Water	21	21	38	55	55	38	48	44	45	51
	Swab	40	40	62	67	79	63	55	57	50	50
Msr	Water	50	50	76	76	76	75	75	76	76	75
	Swab	61	62	76	76	76	76	76	76	76	76
Mph	Water	48	49	76	76	76	76	76	76	75	75

	Swab	54	55	76	76	76	75	76	76	76	76

Supplementary Table S2. Bayesian latent class analysis specificity estimates.

		Sampling Day									
PCR	Sample	0	4	7	14	21	28	35	42	49	56
BVDV	Water	-	-	-	-	-	-	-	-	-	-
	Swab	-	-	-	-	-	-	-	-	-	-
BCV	Water	73	30	21	27	38	50	48	55	45	44
	Swab	62	23	40	33	33	50	55	55	51	38
BRSV	Water	75	54	59	49	39	79	70	76	72	76
	Swab	76	54	52	56	51	60	78	76	72	76
BHV1	Water	75	75	71	61	76	65	76	76	76	70
	Swab	75	76	78	61	76	79	76	76	76	78
HS	Water	75	72	79	70	79	78	78	79	70	67
	Swab	75	72	65	63	55	65	65	65	64	73
MH	Water	76	38	57	56	48	72	77	70	67	60
	Swab	75	38	33	39	41	59	71	63	68	79
PM	Water	75	51	67	56	56	70	78	77	67	65
	Swab	75	39	38	39	44	63	70	71	67	78
MB	Water	76	75	56	62	52	67	79	78	79	70
	Swab	76	76	43	44	39	53	60	65	60	63
Tet	Water	53	54	25	25	25	24	24	28	24	70
	Swab	53	54	25	25	25	24	24	28	25	63
Erm	Water	79	79	62	45	45	62	51	56	56	51
	Swab	60	60	38	33	21	38	44	44	50	50

Msr	Water	50	50	25	25	25	24	25	24	25	24
	Swab	39	39	24	25	25	24	24	24	25	24
Mph	Water	51	51	25	25	25	24	25	24	25	24
	Swab	44	44	25	25	25	25	25	24	25	24
